# Performance and Mechanism of an Alkali-Activated Multifunctional Curing Agent for Silt Solidification

**DOI:** 10.3390/ma19194094

**Published:** 2026-09-24

**Authors:** Wei Zheng, Wei Cui, Si-Xian Zhang, Bei Tang, Jian-Guo Li

**Affiliations:** 1 State Key Laboratory of Hydraulic Engineering Intelligent Construction and Operation, Tianjin University, Tianjin 300072, China; 2 Key Laboratory of Earthquake Engineering Simulation and Seismic Resilience of China Earthquake Administration, Tianjin University, Tianjin 300350, China; 3 STECOL Corporation, Tianjin 300384, China

**Keywords:** solidified silt, alkali activator, multifunctional curing agent, mechanical properties, water stability, microscopic mechanism

## Abstract

In light of its curing and green environmental protection effects, the application of an alkali-activated multifunctional curing agent was undertaken for the remediation of silt in coastal areas. In this paper, the curing effect and curing mechanism of cement–phosphogypsum–fly ash–lime as a multifunctional silt curing agent under the action of an alkaline activator were investigated. The effects of alkaline activator dosing and the relative dosing of a multifunctional curing agent on the mechanical and water stability properties of specimens were comparatively analyzed by carrying out unconfined compressive strength and water stability tests. Furthermore, microanalysis was conducted using scanning electron microscopy and energy spectrum analysis (SEM + EDS), X-ray diffraction (XRD), and thermogravimetric analysis (TG) tests. The objective of these analyses was to reveal the curing mechanism of a multifunctional curing agent. The experimental findings indicate that the addition of an alkaline activator enhances the strength of solidified silt, though it exerts minimal influence on the enhancement of water stability. It is recommended that the mass fraction of NaOH in multifunctional curing agent systems be set at 2%, while the mass fraction of phosphogypsum is advised to be 60%. Under this mixing ratio, the hydration products are able to more effectively act as binding agents for soil particles, thereby filling the internal pores and creating a more compact internal structure in the solidified silt. This, in turn, enhances the compressive strength and water stability of the material. The research results can provide a theoretical basis for the application of industrial solid waste in the treatment of silt and for other practical projects.

## 1. Introduction

In the coastal region of China, a substantial silty soft-ground layer is present. This layer exhibits a range of characteristics, including low strength, high water content, high compressibility, and good mobility. However, it also demonstrates poor bearing capacity and other negative properties [1]. These characteristics indicate that soft ground in areas adjacent to the sea requires specialized treatment and reinforcement in engineering construction to ensure the safety and stability of the structure. The cement mixing pile method is a cost-effective solution for treating and reinforcing soft ground. This method utilizes a deep mixer to blend cement with foundation soil in situ, forming a composite reinforcing body. This process enhances foundation bearing capacity and increases foundation stability. However, given the potential drawbacks associated with the use of cement for curing sludge with high water content, including inadequate early strength, compromised durability, and elevated cement dosage, the exclusive reliance on cement as a curing agent often falls short of delivering optimal engineering and economic benefits. Moreover, the large-scale production and utilization of cement will generate a substantial quantity of carbon dioxide emissions, thereby imposing a considerable environmental strain that is not consistent with the principles of sustainable development [2,3]. Consequently, the imperative to identify a more environmentally sustainable and efficient curing agent that would reduce the amount of cement required has become a pressing concern.

The presence of significant quantities of phosphogypsum and fly ash, resulting from industrial processes, poses a dual challenge: they contribute to environmental degradation and occupy substantial land, thereby leading to resource wastage. In order to realize the resource utilization of industrial solid waste and effectively solve the “three high” problem of common silicate cement, many researchers and scholars have carried out a significant amount of research on the problem of phosphogypsum and fly ash in combination with cement curing in soft soil. Yaghoubi et al. [4,5] utilized fly ash and slag as constituents in the fabrication of geopolymer, a material designed to enhance the properties of soft marine soils. This research demonstrates the efficacy of composite geopolymer as a green cementitious material for the purpose of curing soft soil by means of the deep mixing method. This method is associated with a considerable reduction in environmental impact. By studying the strength and microstructure of fly ash combined with cement solidified soils, Horpibulsuk et al. [6] found that the strength and ductility of solidified soil were greatly enhanced after incorporating fly ash. Zeng et al. [7] employed cement as the primary curing material and industrial waste phosphogypsum as an additive to cure high-water-content silt. The test results showed that the addition of phosphogypsum had significantly improved the strength of solidified soil.

A multitude of studies have demonstrated [8,9,10] that the incorporation of materials with elevated calcium content (e.g., lime, slag, etc.) is advantageous for enhancing the strength development of geopolymers. The presence of calcium has been shown to induce the formation of additional hydrated calcium silicate that coexists with the geopolymer product. This phenomenon has been demonstrated to exert a positive influence on the mechanical properties of the geopolymer material [11], as the soluble phosphorus, fluorine and acid in phosphogypsum inhibit the hydration reaction, to some extent, and prolong the setting time of cement, which reduces the early strength of cement [12]. At the same time, the introduction of sulfur trioxide and some unburnt hazardous components in fly ash can also hinder the hydration of cement. Consequently, the incorporation of lime not only fosters an alkaline milieu conducive to hydration reactions, thereby enhancing geopolymer strength, but also reacts with soluble phosphorus in phosphogypsum to yield phosphate precipitates, thereby augmenting the early strength of the mixture.

In addition, researchers have found that alkali activators promote the reaction of alkali-activated materials, such as fly ash [13]. They also create an alkaline environment that prevents the decomposition of effective hydration products by various types of acids produced by the organic matter in the silt [14]. C.J. Shi et al. [15] concluded that NaOH, Na_2_CO_3_, Na_2_SO_4_, and Na_2_O-NSiO_2_ are the most readily available and economical products. Li et al. [16] used NaOH, Na_2_SO_4_, calcium aluminate cement and γ-nano-Al_2_O_3_, respectively, to stimulate a ternary curing agent consisting of ordinary silicate cement, slag powder and phosphogypsum to cure silt. This study investigated the effect of different excitants on the strength of cured silt, and the results demonstrated that NaOH and Na_2_SO_4_ exhibited excellent excitation effects. Cristelo et al. [17] conducted a study on the compressive properties of alkali-activated fly ash geopolymer-cured clay. The findings indicate that in the presence of an alkali activator, the active silica–alumina components in fly ash, generate silica–aluminate (N-A-S-H) gels, resulting in an unconfined compressive strength of 900 kPa for cured clay.

The performance of alkali-activated soil stabilization systems is strongly dependent on the composition of the precursor and activator, the water content, and mixture proportions. Leong et al. [18] investigated soil–fly ash geopolymer systems and demonstrated that the fly ash/soil ratio, alkali activator content, additional water, and curing conditions all affect strength development. Similar effects have been reported for alkali-activated fly ash-stabilized soils, in which the dosage and composition of the alkaline activator significantly influenced the compressive strength and microstructural development [19,20]. Zeng et al. [7] investigated a cement–slag–phosphogypsum system for marine soft clay and showed that the mechanical performance depended strongly on both the binder composition and the type of chemical activator. These findings indicate that the performance of multi-component stabilization systems cannot generally be attributed to a single mixture variable because the effects of individual constituents are closely coupled. Data-driven approaches have also been increasingly used to quantify the relative importance and interactions of mixture parameters. Kazemi et al. [21], for example, applied machine-learning methods to predict the compressive strength of alkali-activated ultra-high-performance concrete and demonstrated the importance of multiple mixture and curing parameters. Nevertheless, feature importance derived from predictive models represents statistical associations within the available dataset rather than direct evidence of causal mechanisms. Therefore, experimental characterization of both hydration products and microstructural evolution remains necessary for interpreting the underlying mechanisms.

Although previous studies have demonstrated that phosphogypsum, fly ash, lime, and alkaline activators can affect the mechanical performance of stabilized soils, the relationships between mixture composition, water stability, hydration products, and microstructural development in multi-component stabilization systems remain insufficiently understood. In particular, the effects of phosphogypsum and NaOH contents on both mechanical performance and water stability need to be interpreted together with the experimental evidence for hydration products and microstructural characteristics.

Therefore, this study investigates the effects of phosphogypsum and NaOH contents on the unconfined compressive strength and water stability of solidified silt. SEM–EDS, XRD, and TG analyses are further employed to characterize the microstructure, elemental composition, crystalline phases, and thermal decomposition characteristics of the reaction products. By correlating these experimental observations with the macroscopic performance, possible mechanisms responsible for the stabilization behavior are discussed. The research results can provide a reference for the use of industrial waste to solidify silt, which has important economic and environmental benefits.

## 2. Materials and Methods

### 2.1. Materials

The silt sample utilized in this study was obtained from the excavation of a sluice construction project in Zhuhai, Guangdong Province. The undisturbed soil exhibits a gray-brown hue, accompanied by an uneven texture, and contains minor quantities of thin layers of fine sand and shell fragments. The basic physical indicators of undisturbed soil, determined by indoor tests, are shown in Table 1. The chemical composition of the undisturbed soil samples obtained from the project site were determined by X-ray fluorescence (XRF) analysis, and the results are presented in Table 2.

The ordinary silicate cement (P.O42.5) utilized in the experimental study was produced by Huaxin Cement Co. in Yichang, China. Phosphogypsum was supplied by Zhongfu Chemical Group Co., which is based in Wuhan, China. Fly ash was produced by a new material corporation in Tianjin, China. Lime was procured from Komeo Chemical Reagent Co. in Tianjin, China.

The alkaline activator utilized in the experimental procedure was a NaOH solution, supplied by Apex Chemical Reagent Co. in Shanghai, China. It is imperative to note that NaOH exhibits exothermic properties when exposed to water. Therefore, to ensure the accuracy of the experiment, the alkaline activator should be meticulously prepared a minimum of 24 h prior to the commencement of the test.

### 2.2. Sample Preparation and Curing

Data show that the average water content of silt in Zhuhai is 60%, so the initial water content of the soil samples in this test is 60% (i.e., the ratio of water to dry soil mass). The total cementitious material dosage was 30%, by mass of the wet soil. Within the cementitious material, the mass ratio of cement to supplementary cementitious materials (phosphogypsum, fly ash, and lime) was fixed at 3:7. The proportions of phosphogypsum, fly ash, and lime within the supplementary cementitious materials were varied according to the experimental design. The NaOH dosage was set at 1%, 2%, and 3% by mass of the total cementitious material. The specific test program is delineated in Table 3, where PG signifies phosphogypsum, and NH denotes NaOH. The abbreviation “x NH y PG” denotes the x% dosage of NaOH (NH) required to stimulate the y% dosage of phosphogypsum (PG) material for curing in the silt specimen, along with the rest of the analogous material, without further elaboration.

Prior to sample preparation, it is imperative that the retrieved silt undergoes complete desiccation, crushing, and sieving. Firstly, the appropriate mass of dry silt and cementitious materials were weighed, according to the test program. The dry soil and cementitious materials were then poured into a small mixer and thoroughly amalgamated. After that, the weighed distilled water and alkaline activator were successively poured into the beaker and stirred with a glass rod to homogenize the mixture and dissipate heat. Finally, the solution was introduced into the mixture of silt and cementitious materials, and the mixture was stirred for a duration of 10 min to produce samples for testing.

The well-mixed solidified soil was loaded into a standard cubic plastic mold of 50 mm × 50 mm × 50 mm, in replicates of three, and vibrated evenly. To mitigate water evaporation, samples were covered with plastic wrap. Three parallel samples were prepared for each group of samples. The specimens were cured under natural conditions for 48 h and immediately placed into a standard curing box after removal from the mold. The specimens were cured to the design age (28 d) at a temperature of 20 ± 5 °C and a humidity of more than 95%.

### 2.3. Test Methods

#### 2.3.1. Mechanical Performance Test

According to the specification for mix proportion design of cement soil (JGJ-T233-2011) [22], the rate of loading for an unconfined compressive strength test of solidified soil is 1 mm/min. The test instrument employed in this study is an electronic universal material testing machine manufactured by Xinguang Company in Jinan, China.

#### 2.3.2. Water Stability Test

In the context of coastal foundations, cement mixing piles are frequently subject to erosion by water currents, resulting in a deterioration of their performance. Consequently, a comprehensive evaluation of the water stability of solidified soil is imperative. After the specimens had cured in the curing box for 27 d, the specimens were transferred to a container, and distilled water was poured in until the specimens were completely soaked. After 24 h of soaking, the specimens were removed, and the surface water was absorbed with absorbent paper; the unconfined compressive strength test was then carried out. The water stability coefficient is defined as the ratio of the unconfined compressive strength of the soaked and unsoaked samples. A higher water stability coefficient means less loss of strength and better water stability of the solidified soil after soaking.

#### 2.3.3. Microscopic Tests

After 28 d, the broken pieces inside the specimens were removed and dried in an oven at a low temperature of 60 °C. A part of them were ground into powder for XRD and TG tests, and the rest of them, in small fragments with flat sections, were subjected to SEM + EDS tests. The XRD tests were carried out using a Rigaku Smart Lab X-ray diffractometer (Rigaku Corporation, Tokyo, Japan) with a scanning angle ranging from 5° to 75° and a scanning rate of 5 °/min. The SEM + EDS tests were performed with a Hitachi Regulus 8100 field emission scanning electron microscope (Hitachi High-Technologies Corporation, Tokyo, Japan). Prior to its introduction into the SEM instrument, the surface of the specimen should undergo a gold-plating process. This procedure is essential for mitigating the accumulation of charges, which can compromise the integrity of the resulting image. The instrument used for TG tests was a NETZSCH TG 209 F3 Tarsu Thermogravimetric Analyzer (NETZSCH-Gerätebau GmbH, Selb, Germany). The test conditions were as follows: temperature increase rate of 10 °C/min, temperature range of 30–800 °C, and a nitrogen atmosphere. The whole test process is shown in Figure 1.

## 3. Results and Discussions

### 3.1. Unconfined Compressive Strength

As illustrated in Figure 2, with 70% phosphogypsum and 30% fly ash and lime in the multifunctional curing agent, the 28 d strength of the unsoaked specimen was the highest at a 3% mass fraction of NaOH, at 2.77 MPa. The strength was a little lower at a 1% mass fraction, at 2.463 MPa. The lowest recorded strength was achieved at a 2% mass fraction, yielding a result of 1.44 MPa. Subsequent to a 24 h soaking period, the strength of the specimen underwent a gradual decrease, in conjunction with the increasing mass fraction of NaOH. The strength of the 1NH70PG group was reduced to 1.724 MPa, representing a 30% decrease, while the strength of the 3NH70PG group was reduced to 1.336 MPa, indicating a more than 50% decrease.

When the proportion of phosphogypsum is 60% and the proportion of fly ash and lime is 40%, the 28 d strength of the unsoaked specimens increased progressively as the mass fraction of NaOH increased. When the mass fraction was 1%, the strength was 2.194 MPa. A 41.34% increase in strength was observed when the mass fraction was increased to 3%, resulting in a final strength of 3.101 MPa. However, as the mass fraction of NaOH increases, the strength-enhancing effect of the curing agent is diminished. For specimens soaked in water, the strength exhibited a downward trend as the mass fraction of NaOH increased. The most significant reductions in strength were observed at the 2% and 3% mass fractions, while the lowest reductions were observed at the 1% mass fraction.

The 28 d strengths of the unsoaked specimens exhibited minimal variation when the ratio of phosphogypsum to fly ash and lime was 50%. The strength exhibited a maximum and a minimum at a 2% and 1% mass fraction of NaOH, respectively, reaching 2.69 MPa and 2.308 MPa. After the specimens were soaked in water, they underwent a gradual decrease in strength as the mass fraction of NaOH continued to increase. This phenomenon can be attributed primarily to the fact that an excess of NaOH leads to the combination of the OH^−^ group with the metal cations present in the soil. This interaction not only compromises the integrity of the solidified soil structure but also impedes the formation of hydration products and the enhancement of its reinforcing effect [23].

It can also be seen from Figure 2 that when the proportion of phosphogypsum in the multifunctional curing agent is 50%, the average 28 d strength of the unsoaked specimens is 2.451 MPa. When the proportion of phosphogypsum is raised to 60%, the average 28 d strength of the unsoaked specimens is increased to 2.710 MPa. If the proportion of phosphogypsum is increased to 70%, the average 28 d strength of the unsoaked specimens is reduced to 2.224 MPa, a decrease of 17.93%. This may be because the cement hydration reaction, as well as the volcanic ash reaction, of fly ash and the hydration products require an alkaline environment. Excessive phosphogypsum has been observed to compromise the hydration environment of the system, thereby hindering the attainment of optimal conditions for the alkali-stimulated reaction. This, in turn, results in a decline in both the hydration products and the strength of the specimens. It is also possible that the excess phosphogypsum leads to an overproduction of AFt, which causes harmful expansion, leading to cracks in the specimens and a reduction in strength [24].

Subsequent to being immersed in water, the mean 28 d strength of the specimens was determined to be 1.743 MPa, 1.583 MPa, and 1.488 MPa, respectively, at phosphogypsum percentages of 50%, 60%, and 70%. It can be found that as the proportion of phosphogypsum increases, the average strength of the specimens decreases gradually, with a concomitant decrease in the magnitude of the decrease in strength. This is mainly due to the fact that excessive phosphogypsum will show hydrophilicity when encountering water. And the water entering the interior will produce a dissolving effect, destroying the bonding of micro-units between the crystal structure, leading to the softening of structure and affecting its water stability [25].

The above analysis can show that there exists a relatively optimal mixing amount of phosphogypsum and the alkaline activator mass fraction in the multifunctional curing agent. From the overall test results, it is observed that the proportion of phosphogypsum in the multifunctional curing agent is 60%, and the mass fraction of NaOH is 2%, which is more conducive to the enhancement of the strength of solidified soil.

### 3.2. Water Stability Evaluation

The evaluation of the curing effect of a multifunctional curing agent for cement mixing piles in the context of foundation construction in coastal areas encompasses not only the assessment of the compressive strength of solidified soil but also the analysis of its water stability. In this regard, the water stability coefficient is employed as the primary metric for evaluating the water stability of solidified soil. The water stability coefficient is defined as the ratio of the unconfined compressive strength of the soaked and unsoaked soil samples [26], and the calculation formula is shown in Equation (1). The higher water stability coefficient represents the lower strength loss of solidified soil and the increased water stability after soaking. The water stability coefficients of each group of specimens are shown in Table 4.

(1) $$K_{r}=\frac{q_{ui}}{q_{uo}}$$

*K_r_* is the water stability coefficient; *q_ui_* is the unconfined compressive strength of the soaked specimen (MPa); *q_uo_* is the unconfined compressive strength of the unsoaked specimen (MPa).

As illustrated in Table 4, the average water stability coefficient of solidified soil exhibited a gradual decrease in conjunction with the rise in the mass fraction of NaOH. The average water stability coefficients corresponding to the mass fractions of 1%, 2%, and 3% were 0.812, 0.683, and 0.550, respectively. These findings suggest that the alkaline activator did not significantly enhance the water stability of solidified soil. Furthermore, it can also be seen from Figure 3 that the water stability coefficients of the solidified soil exhibit a downward trend across three groups of phosphogypsum occupancies. This finding indicates that merely augmenting the mass fraction of the alkaline activator is insufficient to enhance the water stability of solidified soil.

### 3.3. SEM + EDS Analysis

Scanning electron microscope tests were carried out to further investigate the pore structure and micro-morphology of undisturbed and solidified soil and then to analyze the causes of their strength enhancement. The SEM image of undisturbed soil is shown in Figure 4, and SEM images of a portion of solidified soil are shown in Figure 5. It can be seen in Figure 4 that the undisturbed soil particles are arranged in lamellae, which are not in close contact with each other. This results in soil particles that are susceptible to deformation and relative misalignment under external forces, which is not conducive to strength growth in the absence of cements to keep them linked.

Figure 5a,b show SEM images magnified 1000 and 5000 times. As shown in Figure 5a,b abundant gel-like products and needle- and rod-like crystals were observed in the solidified soil. Based on their characteristic morphologies reported in previous studies [27], these products were assigned to C-S-H and AFt, respectively. Plate-like crystals observed at higher magnification were similarly assigned to CH, based on their typical morphology [23]. The C-S-H gels provide a cementing effect, while the intergrown AFt crystals fill the spaces between soil particles, contributing to the formation of a denser structure.

At higher magnification, Figure 5c,d show that the needle- and rod-like crystals form locally agglomerated structures, accompanied by gel-like hydration products. Some unreacted spherical fly ash particles can also be observed within the pore spaces. These AFt clusters uniformly fill up the pores of silt to form a denser structure. In addition, the gelatinous hydration products are dispersed in and around the AFt clusters, which combine soil particles with AFt. This helps to limit the relative misalignment between AFt and the soil particles, thus forming a stronger structural system. Macroscopically, this manifests itself as an increase in the strength of the solidified soil. From the figure, a small amount of spherical fly ash particles not involved in the reaction can also be seen in the pore space, indicating that this excess portion of fly ash particles was not activated in the alkaline environment provided by NaOH and lime. The microsphere effect of fly ash particles has been demonstrated to reduce porosity and accelerate reactions. However, if the amount of fly ash added is excessive, it can introduce a substantial number of fine particles, thereby altering the size distribution of the cured material [27]. These particles will weaken the cementation force between soil particles, thus reducing the strength of solidified soil. This also explains, from another angle, why the 28 d strength of the unsoaked specimens showed a pattern of first increasing and then decreasing.

To further characterize the elemental compositions of the hydration-product-rich regions, the agglomerated crystal-rich and gel-rich regions observed in Figure 5c,d were further magnified, and EDS area analyses were performed over the selected regions. The results are shown in Figure 6. The analyzed regions mainly contained Ca, Si, O, Al, and S. These elemental compositions are consistent with the presence of calcium–silicate/aluminosilicate gels and sulfate-bearing hydration products. It should be noted that the EDS results represent the average elemental compositions of the selected areas rather than those of individual hydration products [28].

### 3.4. XRD Analysis

To characterize the various types of physical phases of silt cured by multifunctional curing agents, X-ray diffraction tests were carried out on representative specimens. The crystalline phases were identified by comparison with the ICDD Powder Diffraction File (PDF) database, including quartz (PDF#46-1045), muscovite (PDF#07-0042), gypsum (PDF#33-0311), ettringite (PDF#41-1451), brucite (PDF#44-1482), and boracite (PDF#27-0984) [7,16,20]. The identified crystalline phases are shown in Figure 7. From the figure, it can be seen that the peak shape and intensity of the diffraction peaks of the unsoaked and soaked specimens are almost identical, which indicates that there is no change in the physical phase of solidified soil under the influence of soaking. Furthermore, the mineral composition of undisturbed soil primarily consists of quartz, mica, and boracite. A comparison of undisturbed soil and solidified soil specimen reveals discernible alterations in peak shape and intensity, suggesting the occurrence of new mineral formation.

First, the intensity of the diffraction peaks associated with quartz, mica, and boracite decreased in all subgroups. This is partly due to the increased cohesion of soil particles and the formation of flocculated structures that reduce the reflection of incident rays and partly due to the fact that some of the minerals in the soil particles are also involved in the reaction [29]. Secondly, additional diffraction peaks of AFt diffraction appeared at about 2θ = 15° and 2θ = 22.5°, and diffraction peaks of gypsum diffraction appeared at about 2θ = 28°. It is indicated that the addition of the multifunctional curing agent generated a large amount of CH and provided a large amount of Ca^2+^ for the solidified soil. The incorporation of an alkaline activator engenders an alkaline condensation–hardening milieu, a process that facilitates the dissolution of chemical bonds (e.g., Si-O-Si and Al-O-Al) and expedites the hydration reaction. Concurrently, this process effectively stimulates the reactive substances present within the silt [30]. A large number of anions combine with Ca^2+^ to produce gelling substances such as C-S-H and C-A-H. The addition of phosphogypsum also provides Ca^2+^ and SO_4_^2−^ needed for the reaction of the solidified soil, and SO_4_^2−^ further reacts with C-A-H to produce AFt. In addition, since the hydration products N-A-S-H and C-(A)-S-H gel are amorphous substances, there are no obvious characteristic peaks present to allow for the assessment of the intensity. However, the corresponding diffuse peaks can be observed in the range of 2θ = 28°~30°, 33°~35° [31].

### 3.5. TG Analysis

Thermogravimetric analysis tests were carried out on some of the specimens to investigate the effect of the multifunctional curing agent on the amount of reaction product loss on burnout. Figure 8 shows the TG/DTG curves of partially soaked and unsoaked specimens.

The thermogravimetric analysis curve (TG) is the temperature-dependence curve of the mass of samples tested in the thermogravimetric analysis test. From the figure, it can be seen that the mass of each specimen is decreasing due to the dehydration and decomposition of hydration products and carbonates at increasing temperatures. For the unsoaked specimens, the mass loss rates of 2NH70PG, 2NH60PG, and 2NH50PG were, respectively, 9.82%, 13.06%, and 14.20%. It is indicated that the percentage of fly ash and lime has a significant effect on the generation of hydration products in solidified soil. As the percentage of fly ash and lime gradually increases, the generation of hydration products gradually increases, leading to a gradual increase in mass loss. After soaking the specimens in water, the mass loss rates of 2NH70PG, 2NH60PG, and 2NH50PG were 16.84%, 12.16%, and 11.95%. In addition to this, the rate of mass loss of the specimens accelerated, with the 2NH70PG curve decreasing the fastest and the 2NH50PG curve decreasing the slowest. This may be due to the volume expansion caused by the addition of too much phosphogypsum, which not only caused structural damage, but also altered the pore size distribution and pore structure. These alterations increased the water absorption capacity of cured soil. This is consistent with the conclusions obtained from the previous unconfined compressive strength test and once again confirms the existence of a relative optimum in the mixing amount of phosphogypsum with fly ash and lime in a multifunctional curing agent.

In order to highlight the variation in specimen mass with temperature, the TG curve was taken as the first order derivative with respect to temperature, i.e., the differential mass curve (DTG). From the figure, it can be seen that the DTG curves of both soaked and unsoaked solidified soil have more obvious peaks near 80 °C, 130 °C, 400~500 °C and 650 °C. Near 80 °C, the peak is mainly caused by the evaporation of physical binding water and chemical crystallization water [5,12]. By comparing Figure 8a,b, it can be found that the DTG curves of soaked specimens decreased significantly more than did those of unsoaked specimens, which also indicates that the material is gradually losing the binding and crystallization water in this stage. At approximately 130 °C, this phenomenon is predominantly attributable to the decomposition of AFt and the dehydration of C-(A)-S-H [9]. Although the acidity of phosphogypsum inhibits the production of C-(A)-S-H, the production of AFt increases with the increase in the percentage of phosphogypsum, as well as the addition of NaOH. So, the weight loss peak of 2NH70PG is the most pronounced at this stage. In the vicinity of 400~500 °C, the peak is mainly due to the decomposition of calcium hydroxide (Ca(OH)_2_), which loses bound water and produces calcium oxide (CaO) and water vapor (H_2_O) [16]. At temperatures approaching 650 °C, the primary cause of the peak is the decomposition of calcium carbonate (CaCO_3_) [26]. From Figure 8b, it can be seen that as the percentage of fly ash and lime gradually increases, the activated dicalcium silicate and activated tricalcium aluminate in fly ash reacts fully with lime under the activation of NaOH and promotes the generation of a large amount of calcium carbonate. Thus, 2NH50PG exhibits the most obvious weight loss peaks in this temperature range.

### 3.6. Curing Mechanism Analysis

Given the complexity of the cement–phosphogypsum–fly ash–lime system, the exact reaction pathways cannot be conclusively determined from the present experimental results. Based on the SEM–EDS, XRD, and TG observations, together with reaction mechanisms reported in previous studies, several possible processes contributing to the solidification of the silt are discussed, as follows.

The alkaline environment provided by NaOH may promote the dissolution of the vitreous phase in fly ash by facilitating the breaking of Si–O, Al–O, and Ca–O bonds. The released reactive species may subsequently participate in condensation and hydration reactions, contributing to the formation of C-S-H and C-A-H gels [32]. The possible reaction processes are shown in Equations (2) and (3).

(2) $${\mathrm{Si}(\mathrm{OH})}_{4}{+\mathrm{NaOH}+\mathrm{Ca}(\mathrm{OH})}_{2}{+H}_{2}O\to {\mathrm{CaOgSiO}}_{2}{\mathrm{gH}}_{2}O(\mathrm{CSH}\;\mathrm{gel})$$

(3) $${\mathrm{Al}(\mathrm{OH})}_{3}{+\mathrm{NaOH}+\mathrm{Ca}(\mathrm{OH})}_{2}{+H}_{2}O\to {\mathrm{CaOgAl}}_{2}O_{3}{\mathrm{gH}}_{2}O(\mathrm{CAH}\;\mathrm{gel})$$

Ca^2+^ may also contribute to the formation of hydration products. In the multifunctional curing agent system, lime serves as an important calcium source. The hydration of CaO, as shown in Equation (4), provides Ca^2+^ and OH^−^, which may promote the formation of Ca-containing hydration products, while maintaining an alkaline environment [33]. Meanwhile, CaSO_4_ in phosphogypsum provides additional Ca^2+^ and SO_4_^2−^, and the sulfate may react with Al-containing species to form AFt, as represented by Equation (5) [34].

(4) $${\mathrm{CaO}+H}_{2}O\to {\mathrm{Ca}}^{2+}{+2\mathrm{OH}}^{-}$$

(5) $${3\mathrm{CaSO}}_{4}{+3\mathrm{CaOgAl}}_{2}O_{3}{+32H}_{2}O\to {3\mathrm{CaOgAl}}_{2}O_{3}{g3\mathrm{CaSO}}_{4}{g32H}_{2}O\left(\mathrm{Ettringite}\right)$$

Ion exchange and adsorption may further contribute to the stabilization of the silt. Ca^2+^ released from the cementitious materials may exchange with Na^+^ or K^+^ associated with the soil particles, reducing the thickness of the diffuse double layer and promoting particle aggregation [35,36]. The proposed ion exchange and adsorption process is illustrated in Figure 9. Water immersion may weaken the stabilized structure through several processes. Water penetration may weaken interparticle bonding, expand the diffuse layer of soil particles, and promote the dissolution or deterioration of some cementitious products, which could contribute to the observed reduction in strength [37].

Overall, the experimental observations suggest that the improvement in the solidified silt may result from the combined effects of alkaline activation, hydration-product formation, physical filling, and ion exchange. The reaction pathways discussed above represent possible mechanisms inferred from the present experimental evidence and previous studies rather than definitive reaction pathways.

## 4. Conclusions

This paper focuses on the performance and curing mechanism of a multifunctional curing agent consisting of cement–phosphogypsum–fly ash–lime-cured silt under the action of an alkaline activator. The mechanical properties and water stability were studied using an unconfined compressive strength test and a water stability test. And the curing mechanism was analyzed by means of SEM, XRD and other microscopic tests. The following conclusions are obtained:

(1) In the multifunctional curing agent system, an increase in the percentage of phosphogypsum was observed to result in an initial increase, followed by a subsequent decrease, in the average strength of the unsoaked specimens. This observation indicates that the incorporation of phosphogypsum enhances the reaction of lime and fly ash, resulting in the formation of cementitious material and AFt. This material is capable of filling the internal pores of the soil, thereby enhancing its strength upon solidification. However, if too much phosphogypsum is added, harmful expansion will occur and result in cracks and reduced strength of the specimen. Therefore, it is recommended that the proportion of phosphogypsum in the multifunctional curing agent is 60%.
(2) The alkaline activator facilitates the breaking of chemical bonds in fly ash vitreous and accelerates the hydration reaction, promoting the generation of hydration products. However, the results of the water stability test showed that the strength and water stability coefficient of the specimens showed a gradual decrease with the gradual increase in the mass fraction of NaOH. It can be inferred that the alkaline activator has a limited effect on the enhancement of water stability. Therefore, in order to ensure that the alkaline stimulant can promote the reaction, while reducing the negative impact of high alkalinity, the recommended NaOH mass fraction is 2%.
(3) The results of the SEM + EDS test showed that the multifunctional curing agent has a good cementing and filling effect on silt, increasing the contact area of particles. Meanwhile, the microsphere effect of fly ash can also reduce the porosity, which makes the soil particles arranged in a lamellar body difficult to deform. However, the introduction of a large number of fine fly ash particles will result in some of these particles remaining inactivated, thus changing the particle size distribution of the cured material. Consequently, the friction and cementation between soil particles are reduced, which is macroscopically manifested as a reduction in the strength of the solidified soil.
(4) With the gradual increase in the proportion of fly ash and lime, the mass loss rate of the unsoaked specimens gradually increases, and the mass loss rate of the soaked specimens gradually decreases. This indicates that the relative content of phosphogypsum with fly ash and lime in a multifunctional curing agent has a greater influence on the generation of hydration products and the change in pore structure. The appropriate mixing ratio can not only promote the densification of the structure of solidified soil, but also prevent structural damage due to excess AFt, which ensures the strength and water stability of solidified soil.

## Figures and Tables

**Figure 1:**
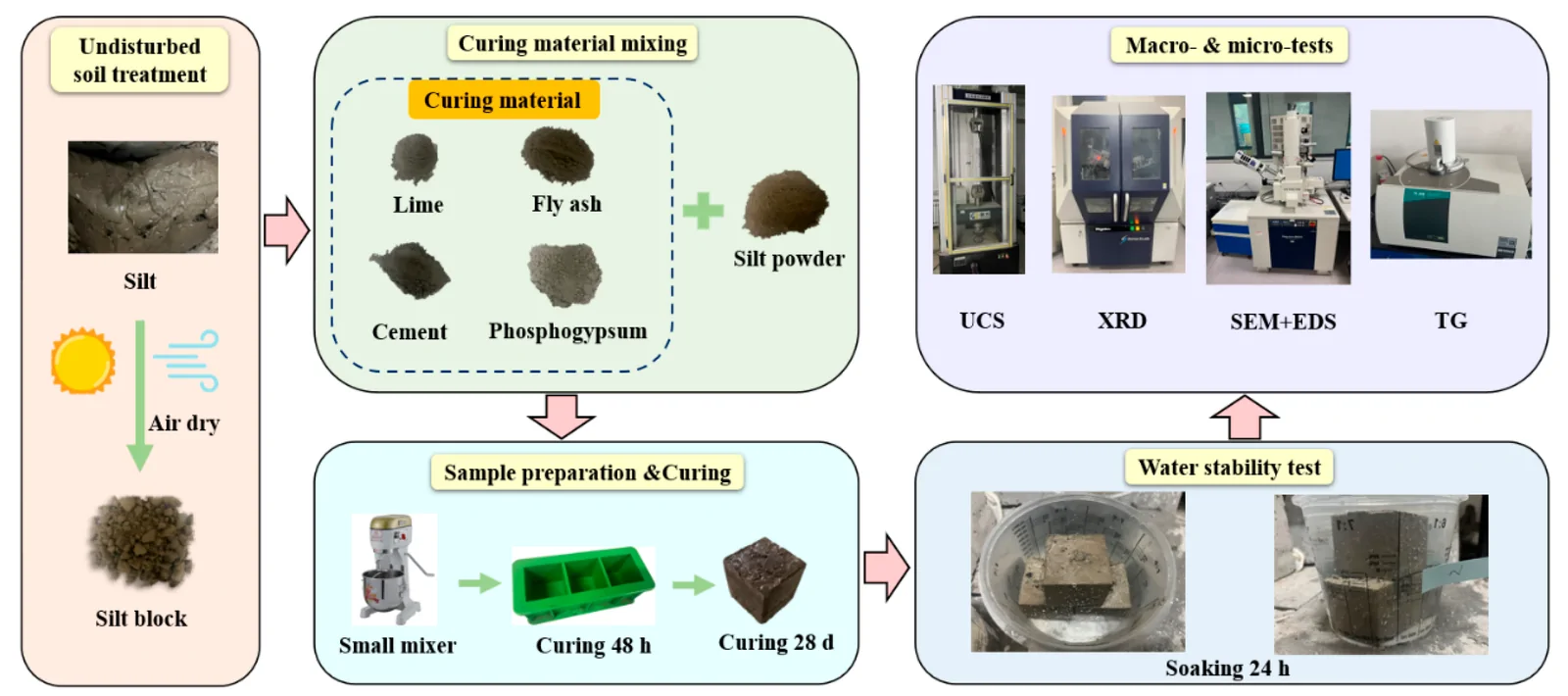
Schematic diagram of the test process and equipment.

**Figure 2:**
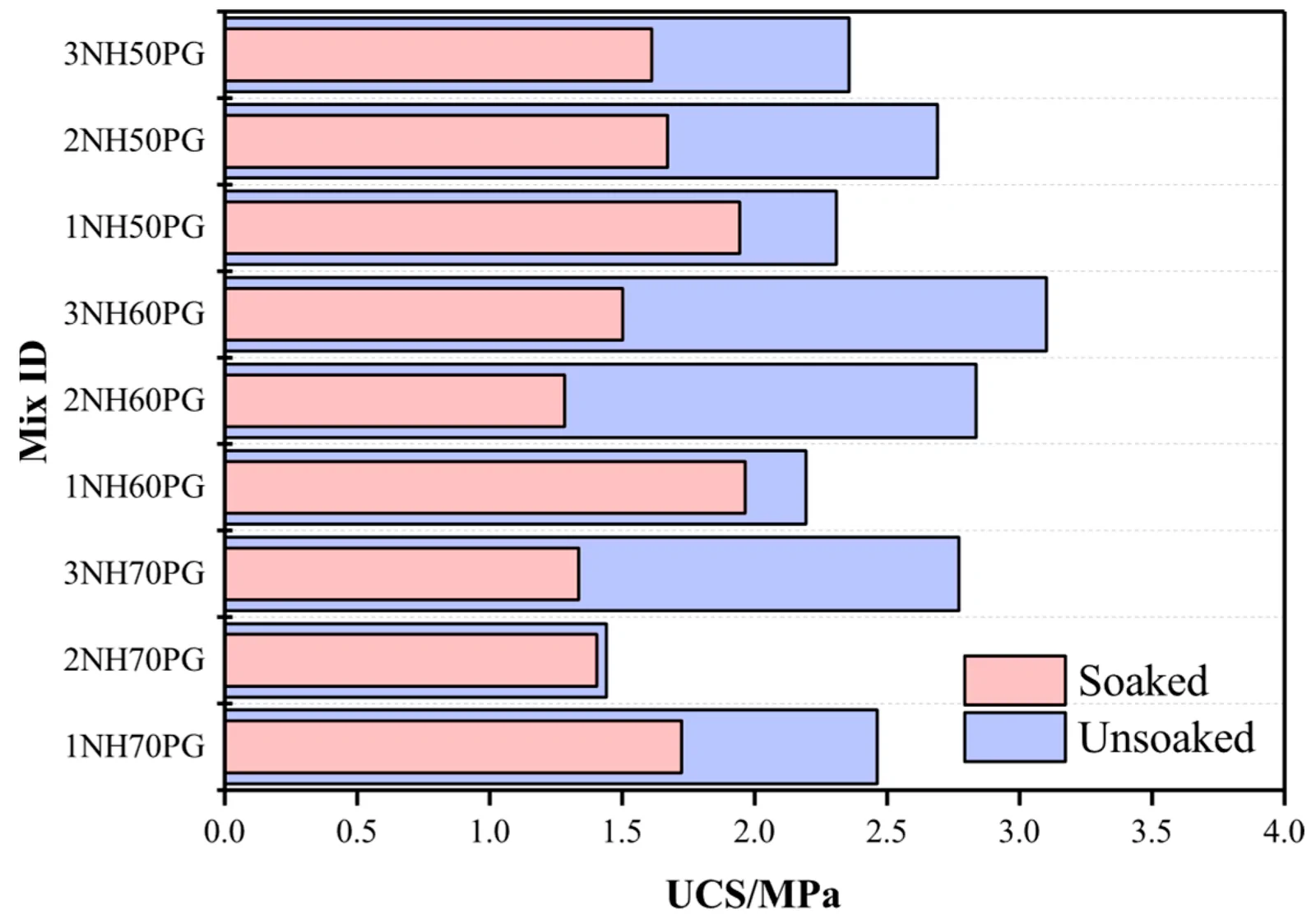
Compressive strength of solidified soil specimens (28 d).

**Figure 3:**
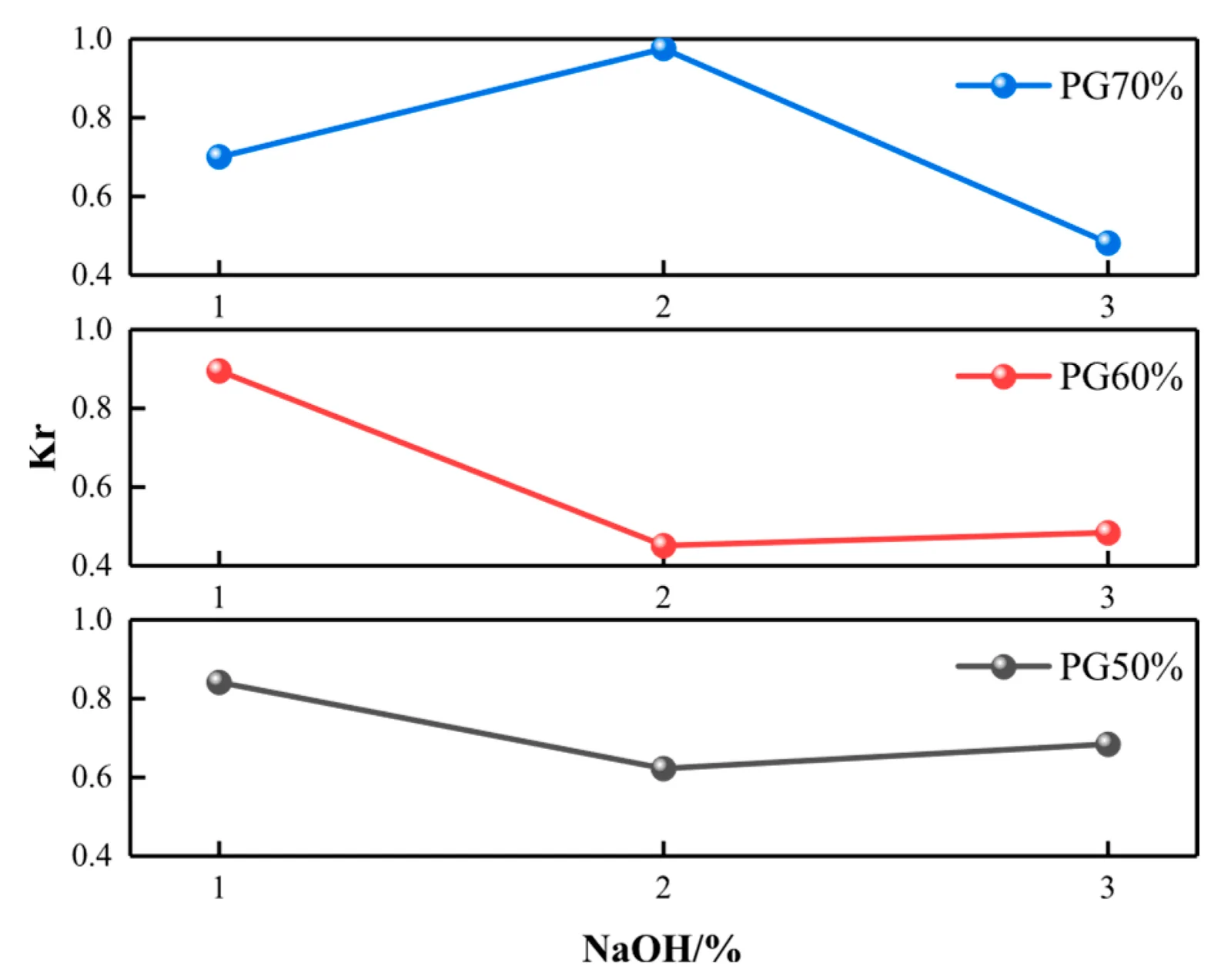
Curves of water stability coefficient.

**Figure 4:**
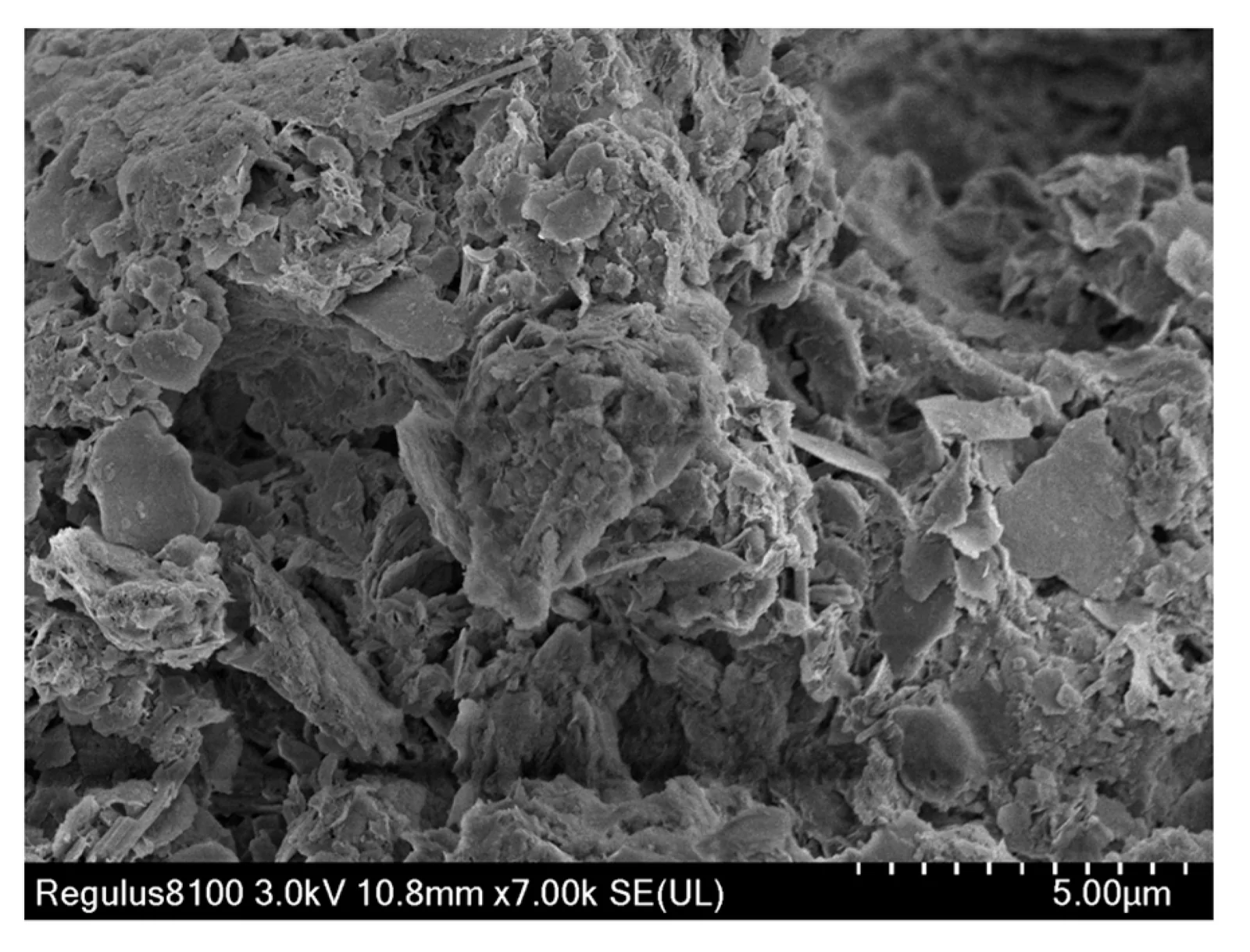
SEM image of undisturbed soil.

**Figure 5:**
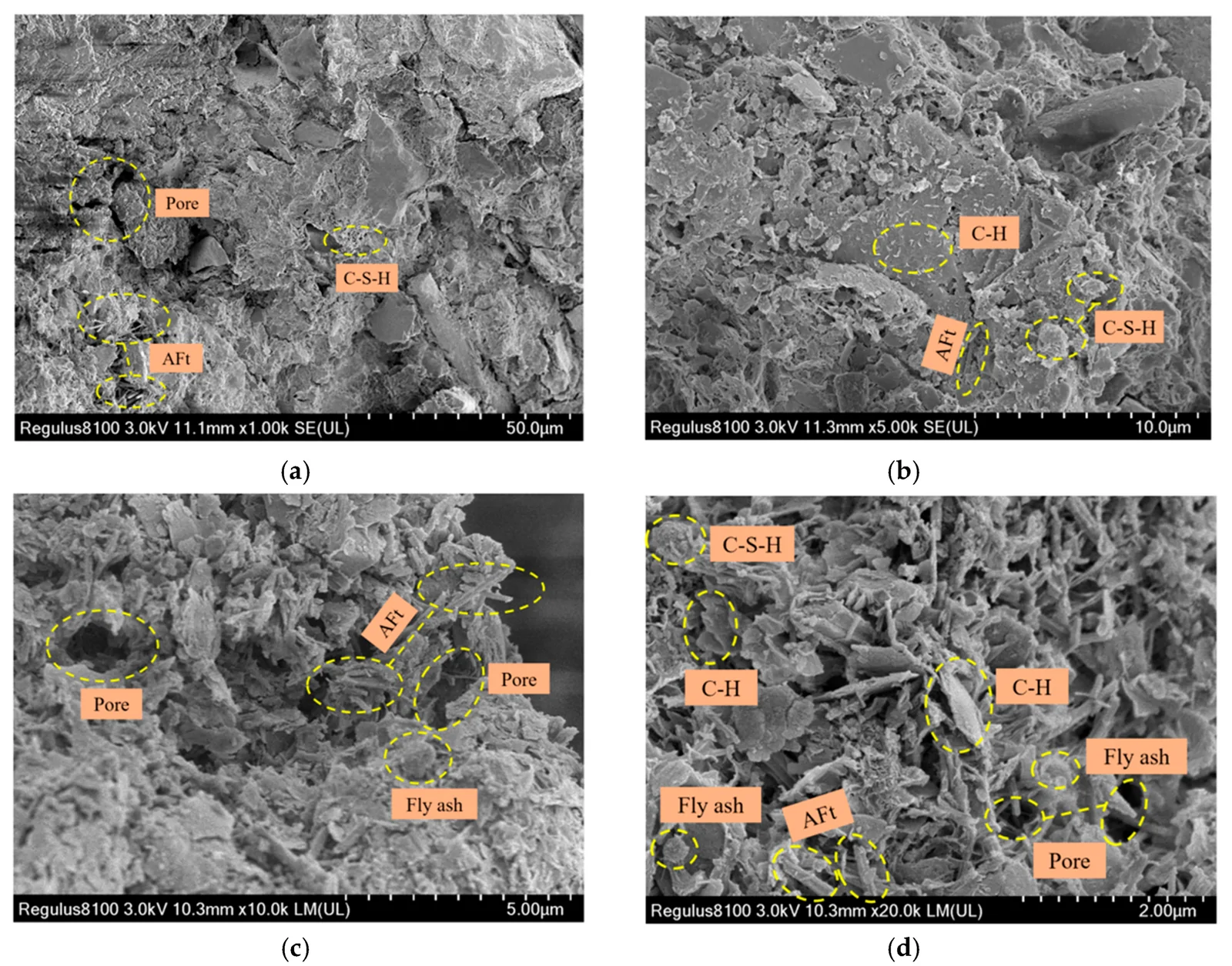
SEM images of solidified soil (28 d). (**a**) ×1000, (**b**) ×5000, (**c**) ×10,000, (**d**) ×20,000.

**Figure 6:**
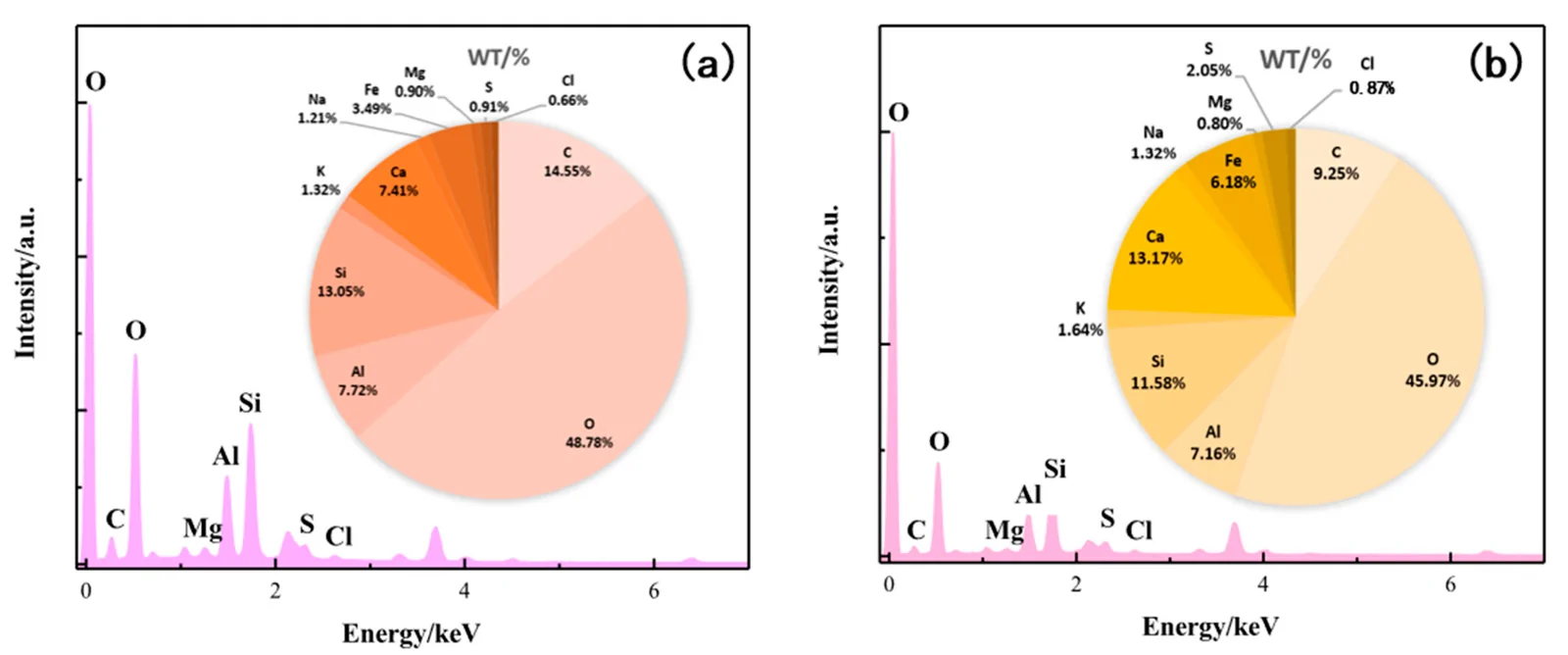
Results of EDS analysis (28 d): (**a**) location c; (**b**) location d.

**Figure 7:**
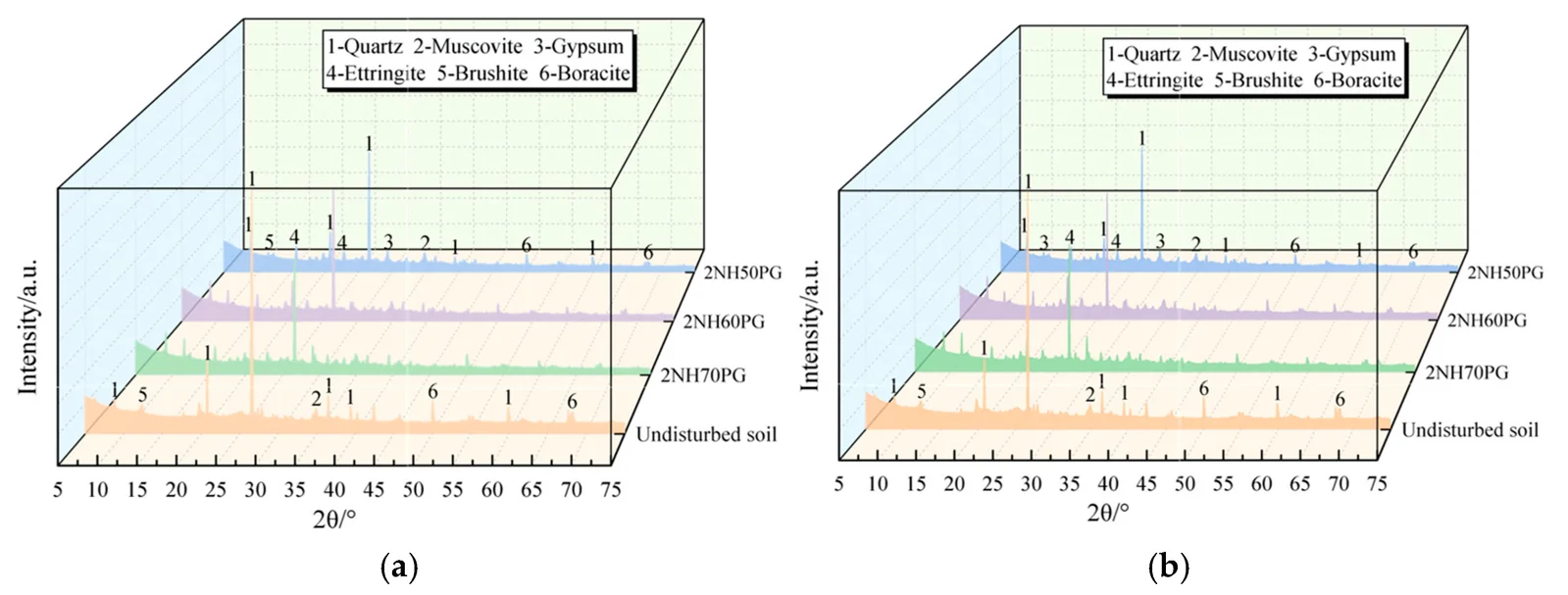
XRD diffraction pattern of some specimens (28 d): (**a**) unsoaked; (**b**) soaked.

**Figure 8:**
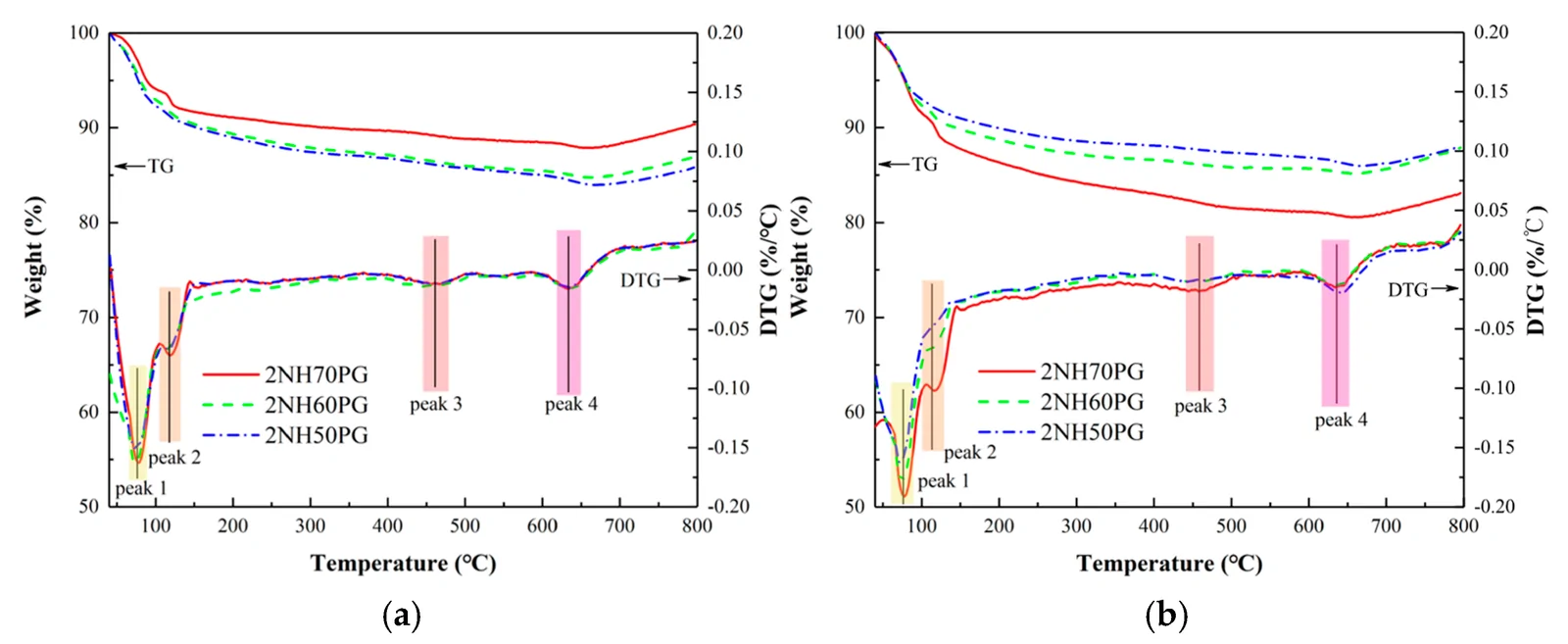
TG/DTG curves of solidified soil specimens (28 d): (**a**) unsoaked; (**b**) soaked.

**Figure 9:**
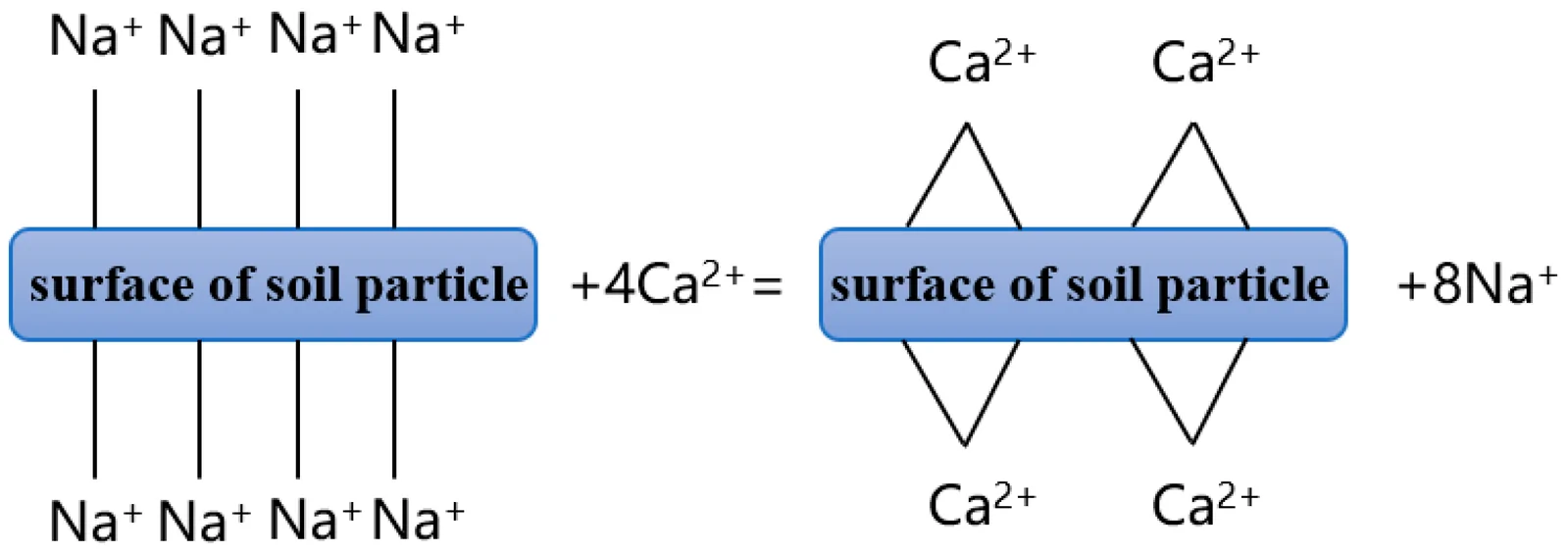
Schematic diagram of ion exchange and adsorption.

**Table 1:** Indicators of physical properties of the test soil.

Test Soil	Water Content ω	Specific Gravity $G_{s}$	Wet Density ρ	Dry Density $\rho_{d}$	Saturation $\rho_{d}$	Porosity Ratio e	Liquid Limit $\omega_{L}$	Plastic Limit $\omega_{P}$	Plasticity Index $I_{P}$	Liquidity Index $I_{L}$
	(%)		(g/cm^3^)	(g/cm^3^)	(g/cm^3^)		(%)	(%)		
Silt	64.5	2.65	1.59	0.972	98.2	1.739	49.8	26.8	23.0	1.63

**Table 2:** Chemical composition content (g/kg).

	CO_3_^2−^	HCO_3_^−^	SO_4_^2+^	Cl^−^	Ca^2+^	Mg^2+^	Na^+^	K^+^	Organic Mass (%)
Silt	<0.007	0.373	0.399	3.381	0.096	0.017	2.405	0.107	2.14

**Table 3:** Test program (%).

Mix ID	Cement	Phosphogypsum	Lime + Fly Ash (Mass Ratio of Lime to Fly Ash 2:1)	NaOH
1NH70PG	30	49	21	1
2NH70PG	30	49	21	2
3NH70PG	30	49	21	3
1NH60PG	30	42	28	1
2NH60PG	30	42	28	2
3NH60PG	30	42	28	3
1NH50PG	30	35	35	1
2NH50PG	30	35	35	2
3NH50PG	30	35	35	3

**Table 4:** Water stability coefficient of solidified soil specimens (28 d).

Mix ID	Water Stability Coefficient $K_{r}$
1NH70PG	0.700
2NH70PG	0.975
3NH70PG	0.482
1NH60PG	0.895
2NH60PG	0.452
3NH60PG	0.484
1NH50PG	0.842
2NH50PG	0.622
3NH50PG	0.684

## Data Availability

The data presented in this study are available on request from the corresponding author due to legal reasons.
